# Screening of Potential Biomarkers for Gastric Cancer with Diagnostic Value Using Label-free Global Proteome Analysis

**DOI:** 10.1016/j.gpb.2020.06.012

**Published:** 2021-02-17

**Authors:** Yongxi Song, Jun Wang, Jingxu Sun, Xiaowan Chen, Jinxin Shi, Zhonghua Wu, Dehao Yu, Fei Zhang, Zhenning Wang

**Affiliations:** Department of Surgical Oncology and General Surgery, Key Laboratory of Precision Diagnosis and Treatment of Gastrointestinal Tumors, Ministry of Education, The First Affiliated Hospital of China Medical University, Shenyang 110001, China

**Keywords:** Gastric cancer, Proteomics, Label-free, Bioinformatics, Diagnosis

## Abstract

**Gastric cancer** (GC) is known as a top malignant type of tumors worldwide. Despite the recent decrease in mortality rates, the prognosis remains poor. Therefore, it is necessary to find novel biomarkers with early diagnostic value for GC. In this study, we present a large-scale proteomic analysis of 30 GC tissues and 30 matched healthy tissues using **label-free** global proteome profiling. Our results identified 537 differentially expressed proteins, including 280 upregulated and 257 downregulated proteins. The ingenuity pathway analysis (IPA) results indicated that the sirtuin signaling pathway was the most activated pathway in GC tissues whereas oxidative phosphorylation was the most inhibited. Moreover, the most activated molecular function was cellular movement, including tissue invasion by tumor cell lines. Based on IPA results, 15 hub proteins were screened. Using the receiver operating characteristic curve, most of hub proteins showed a high diagnostic power in distinguishing between tumors and healthy controls. A four-protein (*ATP5B*-*ATP5O*-*NDUFB4*-*NDUFB8*) diagnostic signature was built using a random forest model. The area under the curve (AUC) values of this model were 0.996 and 0.886 for the training and testing sets, respectively, suggesting that the four-protein signature has a high diagnostic power. This signature was further tested with independent datasets using plasma enzyme-linked immune sorbent assays, resulting in an AUC value of 0.778 for distinguishing GC tissues from healthy controls, and using immunohistochemical tissue microarray analysis, resulting in an AUC value of 0.805. In conclusion, this study identifies potential biomarkers and improves our understanding of the pathogenesis, providing novel therapeutic targets for GC.

## Introduction

Gastric cancer (GC) is one of the most common malignant, aggressive tumors, causing approximately 723,100 deaths worldwide in 2012 [1], particularly in East Asia [2]. It is a complex disease with histological and etiological heterogeneity [3]. Large genomic variations have been detected in GC patients [4]. A number of patients are diagnosed with GC at a later phase because of asymptomatic nature of the disease [5]. Despite a decrease in mortality rates in recent years, GC prognosis remains at the poor progress, with only 28.3% of new cases can survive for more than 5 years [6]. Our understanding of GC pathogenesis and molecular biology has improved, yet it is still necessary to identify novel biomarkers with early diagnostic value, to determine efficient diagnostic methods, and to discover new targets for treating GC.

Cancer development and progression require molecular alterations at multiple levels including the genome, transcriptome, proteome, and metabolome [7]. In the past decade, numerous studies have examined molecular mechanisms of cancer using genomic and transcriptomic analyses. Protein dynamics are crucial for determining cancer phenotype, and the rapid development of quantitative proteomic approaches for studies on cancer proteomics stimulated investigations characterizing proteogenomic landscapes for many human cancers, including colorectal cancer [8], prostate cancer [9], breast cancer [10], lung adenocarcinoma [11], and ovarian cancer [12]. These efforts promoted the use of mass spectrometry (MS)-based proteogenomics for clinical use [13].

Several recent studies have investigated proteomic aspects of GC. Using the Isobaric Tags for Relative and Absolute Quantitation (iTRAQ) method, integrated with high-resolution MS analysis, a previous study identified 3914 different proteins in six biopsies from different disease stages ranging from chronic gastritis and intestinal metaplasia to gastric adenocarcinoma [14]. Another study examined four GC tissues and four adjacent normal tissues and identified 431 differentially expressed proteins (DEPs) using iTRAQ-based quantitative proteomic analysis [15]. This study showed correlations between MTA2 and HDAC1 expression levels and between lymph node metastasis and tumor‐node‐metastasis (TNM) staging for GC.

One major limitation of current GC proteomics studies is sample size. Small sample sizes introduce bias to findings and result in data inconsistencies. Also, due to individual heterogeneity, paired tumor and healthy control samples from the same patient should ideally be compared when searching for proteomic alterations [16]. Additionally, integrating proteomics and robust bioinformatics methods might help identify potential novel biomarkers with diagnostic power for GC.

In this study, we present a large-scale proteomic analysis of 30 GC tissues and 30 matched healthy tissues using label-free global proteome profiling. This proteomic analysis helped identify 537 DEPs, including 280 upregulated and 257 downregulated proteins. Results of the ingenuity pathway analysis (IPA) indicated that the sirtuin signaling pathway was most activated, whereas oxidative phosphorylation was the most inhibited pathway. Moreover, the most activated molecular function (MF) was cellular movement, including tissue invasion by tumor cell lines. Subsequently, 15 hub proteins were screened based on IPA enrichment results. Using the receiver operating characteristic (ROC) curve, most of these hub proteins had reliable diagnostic potential to distinguish between tumors and healthy controls. After that, a four-protein (*ATP5B*-*ATP5O*-*NDUFB4*-*NDUFB8*) diagnostic signature was built using a random forest model. The area under curve (AUC) value of this model was 0.886 for the testing set, suggesting a high diagnostic potential. Additional independent datasets were used to test our four-protein signature by plasma enzyme-linked immune sorbent assays (ELISA), which yielded an AUC value of 0.778 and accuracy of 71.8% to distinguish GC from healthy controls, and by immunohistochemical tissue microarray analysis, which yielded an AUC value of 0.805. In conclusion, this study identified highly dysregulated proteins and potential biomarkers with potential use in detecting GC. These results further improve our understanding of GC pathogenesis and identify novel and specific therapeutic targets for this cancer.

## Results

### Global proteome profiling in the GC cohort

**Figure 1:**
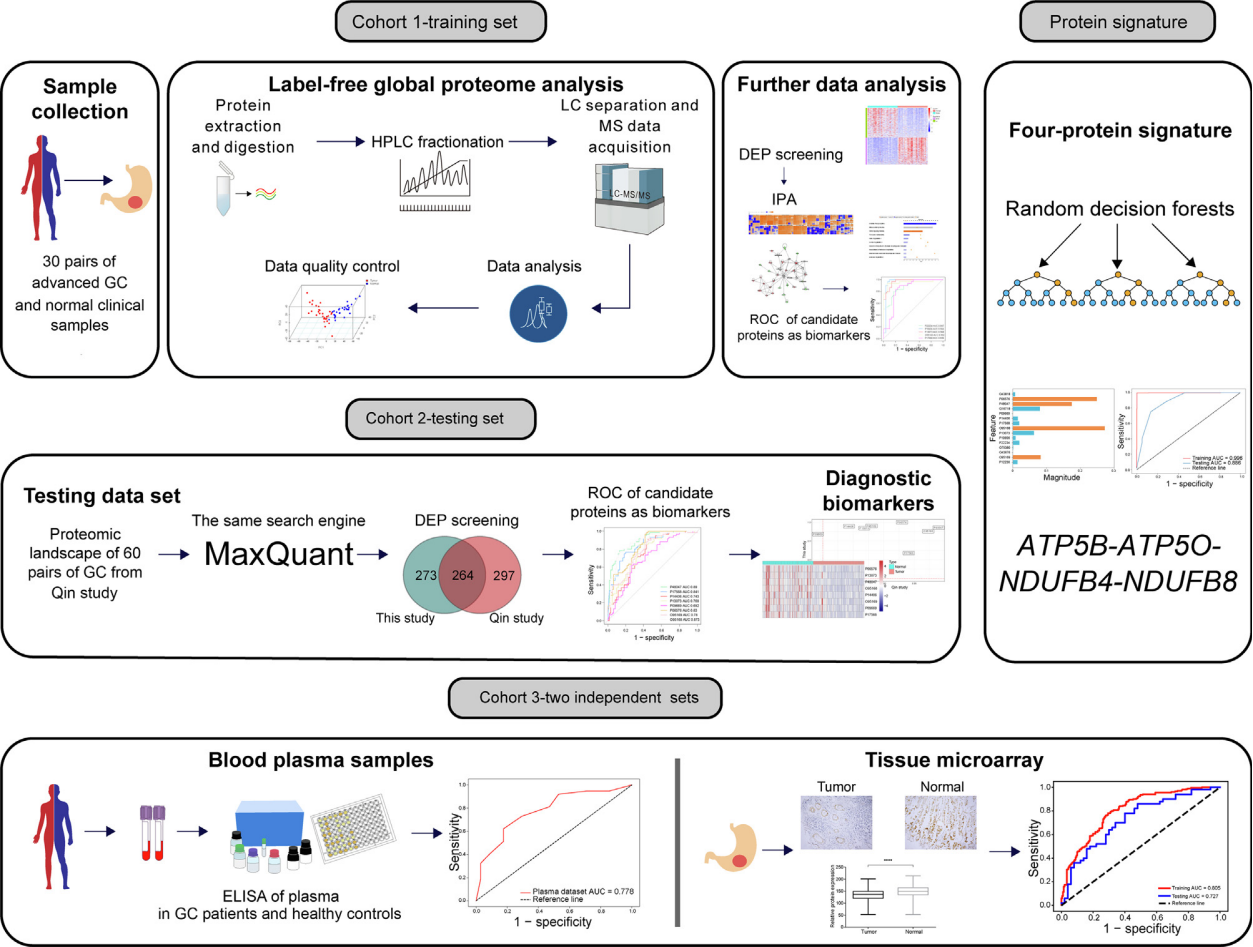
**Workflow of the study** Screening of potential biomarkers for GC with diagnostic value using label-free global proteome analysis. GC, gastric cancer; LC, liquid chromatography; HPLC, high performance liquid chromatography; MS, mass spectrometry; DEP, differentially expressed protein; IPA, ingenuity pathway analysis; ROC, receiver operating characteristic; ELISA, enzyme-linked immune sorbent assay.

**Supplementary Figure S1:**
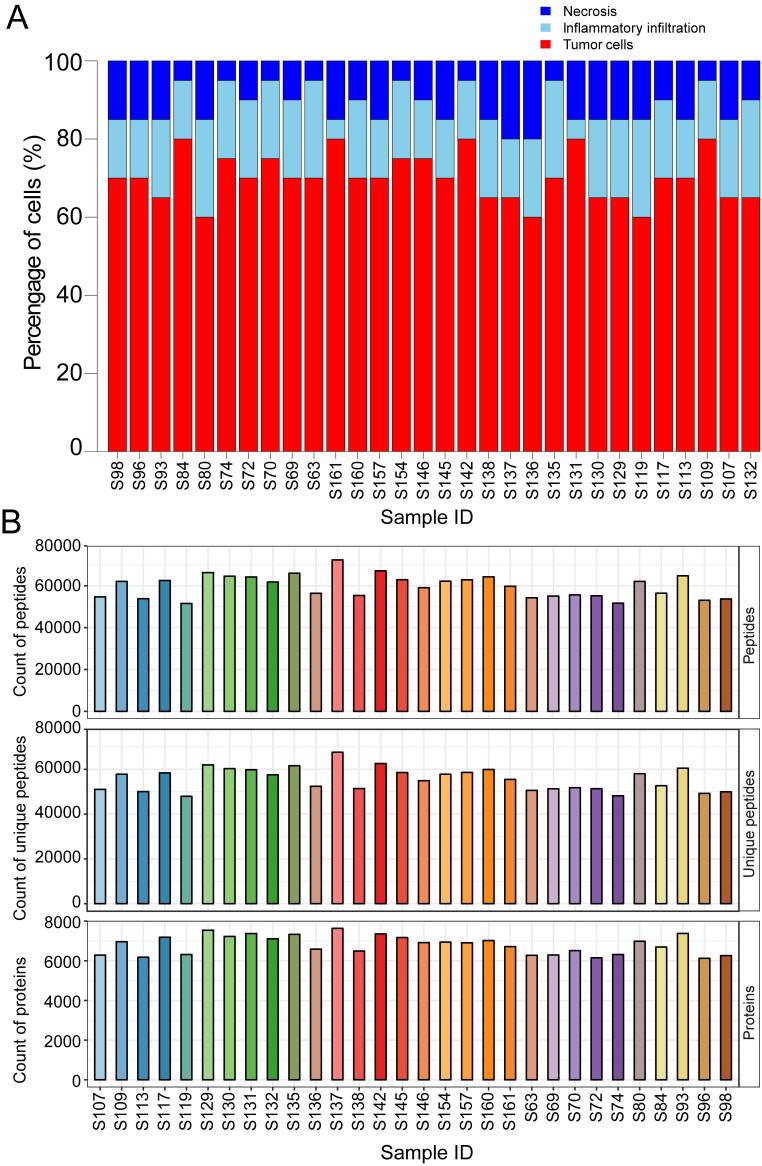
**Quality control of clinical samples and global proteome profiling**. **A**. The neoplastic purity analysis of all 60 samples. **B**. The distribution of peptides, unique peptides, and identified proteins in this study.

**Supplementary Figure S2:**
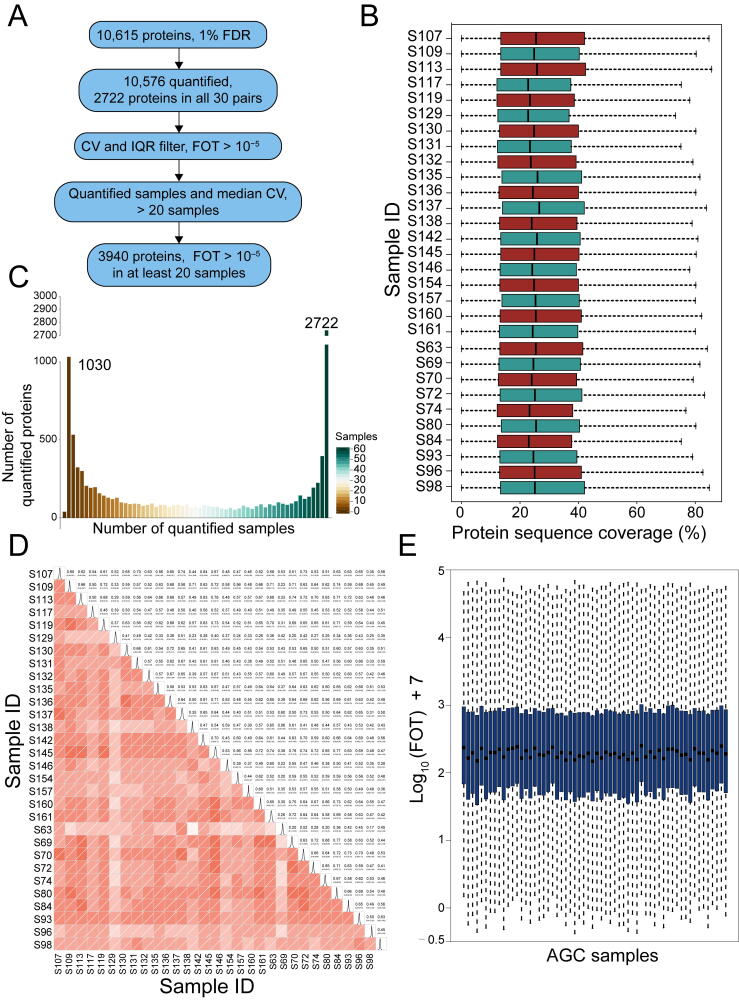
**General overview of global proteome profiling**. **A**. Proteomic data filtered at different levels. FDR, false discovery rate; CV, coefficients of variation; IQR, interquartile range; FOT, fraction of total. **B**. Distribution of the protein sequence coverage across all 60 samples from 60 gastric cancer patients. **C**. Distribution of quantified proteins across all 60 samples. **D**. Spearman’s correlation coefficient of the protein ratios in 30 GC tissues and 30 matched healthy tissues. **E**. Distribution of log10(FOT)+7 of the identified proteins in 30 GC tissues and 30 matched healthy tissues. Boxes range from the lower quartile to the upper quartile. GC, gastric cancer.

In this study, we conducted an integrated analysis of the global proteome profile of GC (Figure 1). Thirty primary tumor tissues and corresponding adjacent healthy tissues were obtained after surgical resection from a total of 30 GC patients at the First Hospital of China Medical University. The neoplastic purity analysis of all 60 samples is shown in Figure S1A and Table S1. High-resolution liquid chromatography-tandem MS (LC-MS/MS) was used to identify differences in proteomic profiles between the tumor and healthy samples. LC-MS/MS analyses were performed using MaxQuant software (v1.4.1.2) [17]. The distributions of peptides, unique peptides, and identified proteins are shown in Figure S1B. We used the label-free quantification (LFQ) algorithm embedded in MAXQUANT to quantify protein expression, and peptide-spectrum matching, false discovery rate (FDR), peptide FDR threshold, and protein FDR threshold were all set to 1%. A total of 10,615 proteins were identified in this study, with an average protein coverage rate of 28% (Figure S2A and B). Of these, expression of 10,576 proteins was quantified, with expression of 2722 proteins quantified across all 30 tissue pairs (Figure S2C).

We began by calculating the protein ratio of tumor *versus* healthy tissues (Log_2_ T/N ratio) for one paired sample using LFQ values. After that, we generated Spearman’s correlation coefficient matrices for all 30 patients using protein ratios (Figure S2D). The fraction of total (FOT) value was used to determine the distribution of protein expression across all GC samples (Figure S2E). Our results indicated consistent proteome identification and quantification throughout our study.

**Supplementary Figure S3:**
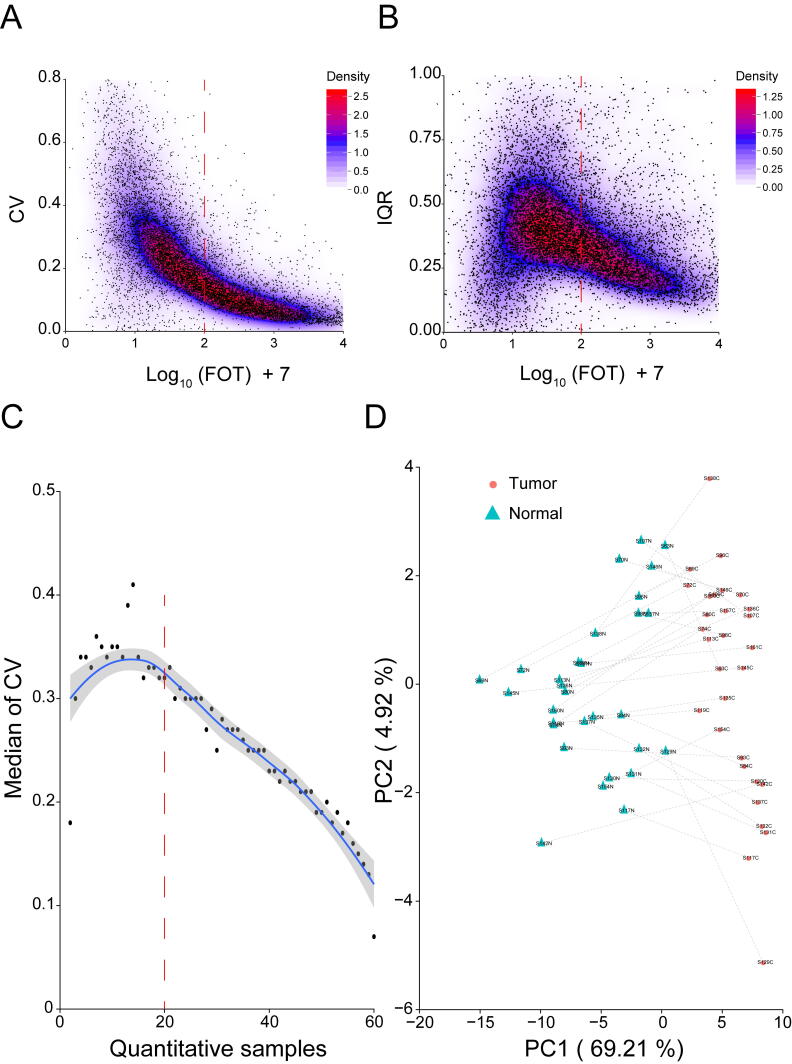
**General overview of proteome quantification in GC**. **A**. Relationship between CV and log10(FOT)+7. When FOT reached more than 10-5, overall CV significantly decreased. **B**. Relationship between IQR and log10(FOT)+7. The increased performance of IQR was discontinued after the FOT reached > 10-5. **C**. Relationship between quantitative samples and median CV. When quantitative samples reached > 20, median CV significantly decreased. **D**. PCA analysis showing tumor and matched healthy tissues. PC, principal component. CV, coefficient of variance. IQR, interquartile range. FOT, fraction of total.

We next determined the coefficient of variation (CV) and interquartile range (IQR) of the proteins. The overall CV decreased significantly when the FOT value was higher than 10^−5^ (Figure S3A). Additionally, the increased performance of the IQR was discontinued when the FOT value was higher than 10^−5^ (Figure S3B), suggesting that the most suitable value for accurate quantification was when the FOT value was 10^−5^. This result is consistent with the cut-off value from a previous GC proteomics study [16]. We also calculated the distribution of the quantitative samples and found a median CV of *(Log_10_ FOT)* + 7. The median CV was significantly decreased when the number of quantitative samples was over 20 (Figure S3C). Therefore, we filtered 3940 proteins using the above cut-off value and the number of quantitative samples (Table S2). For these proteins, we used principal component analysis (PCA) to analyze expression in tumor tissues and corresponding matched healthy tissues (Figure S3D). The proportions of variance of PC1 and PC2 were 69.21% and 4.92%, respectively. These PCA results indicate a clear distinction between the proteomes of tumor and healthy tissues.

### Identification of DEPs in GC

**Figure 2:**
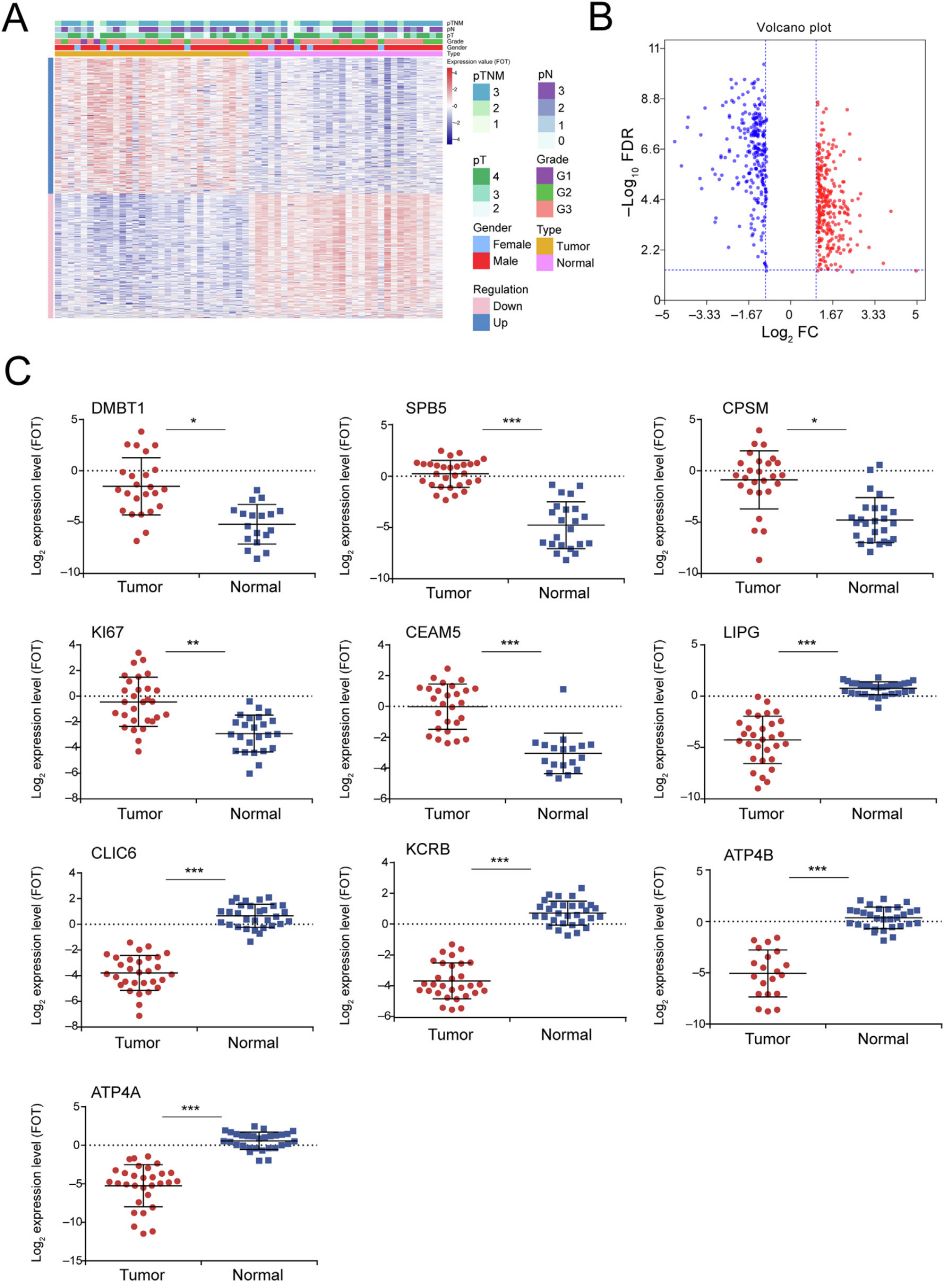
**Proteomic features ofDEPs inGC** **A.** Heatmap of DEPs in GC. **B.** A volcano plot for DEPs. The differential expression ratio of Log_2_ FC (*x* axis) and the −Log_10_ FDR value (*y* axis) were plotted for each identified protein. **C.** Expression profiles of the top ten significant DEPs in tumor and normal tissues. *, *P* < 0.05; **, *P* < 0.01; ***, *P* < 0.001 (paired-samples *t*-test). pT, pathological tumor stage; pN, pathological lymph node stage; pTNM, pathological tumor‐node‐metastasis stage; FC, fold change; FDR, false discovery rate; FOT, fraction of total.

**Figure 3:**
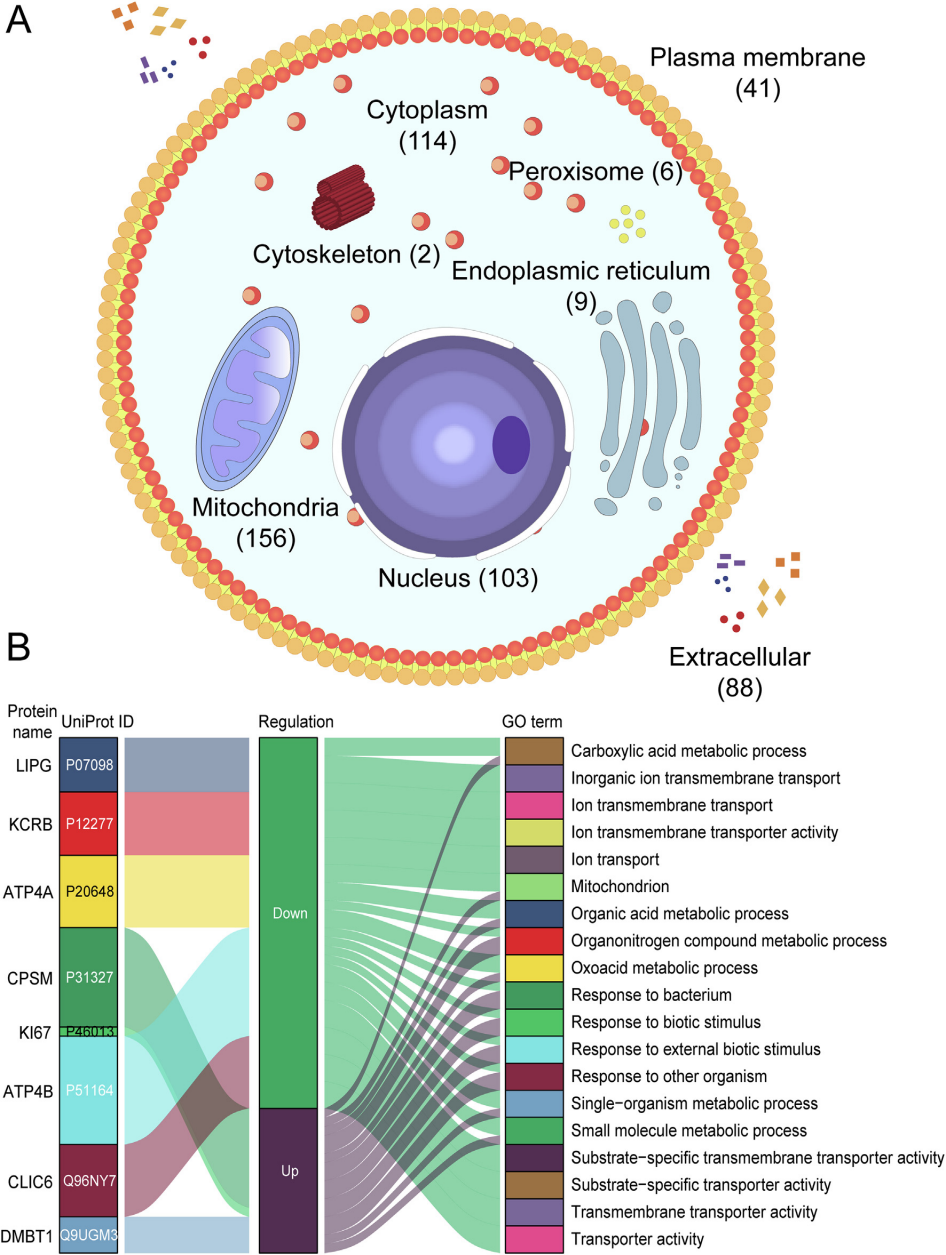
**Subcellular distribution and GO term enrichment analysis ofDEPs inGC** **A.** Subcellular distribution of DEPs. **B.** Graphical summary of DEPs and GO term enrichment analysis. GO, gene ontology.

We next assessed significant quantitative differences between tumor tissues and matched healthy tissues. DEPs were screened using a filter criterion of |Log_2_ fold change| > 1 and FDR < 0.05. This identified 537 DEPs, including 280 upregulated and 257 downregulated proteins (Figure 2A; Table S3). A volcano plot showing statistically significant DEPs between tumor and healthy tissues was constructed (Figure 2B). Expression levels of the top ten significant DEPs, DMBT1, SPB5, CPSM, KI67, CEAM5, ATP4A, ATP4B, CLIC6, KCRB, and LIPG, are shown in Figure 2C and Table 1. According to subcellular localization analysis, a good many DEPs got annotation as mitochondrial, suggesting that the GC proteome is involved in tumor energy metabolism (Figure 3A). The gene ontology (GO) term enrichment analysis (Table S4) revealed that upregulated DEPs in GC were significantly enriched in activities of the nucleolus, DNA, and RNA. Downregulated DEPs were mostly enriched in mitochondrial processes, cellular respiration, and oxidative phosphorylation (Figure 3B).

**Table 1:** **The information of toptensignificant DEPs**

### Proteomic pathways and potential hub proteins in GC

**Figure 4:**
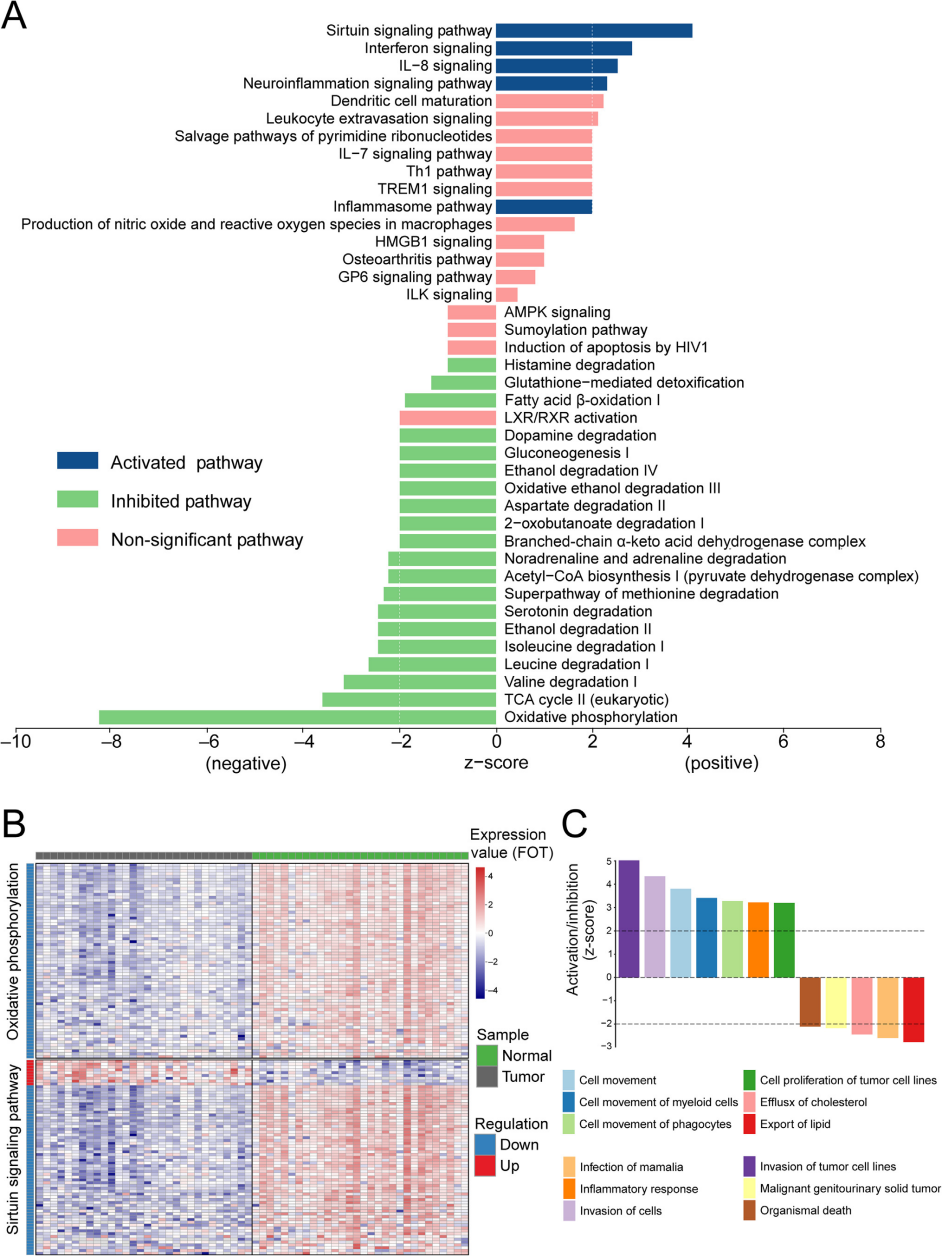
**Activated or inhibited pathways and potential hub proteins inGCusingIPA** **A.** Canonical pathway analysis of DEPs. **B.** Expression profiles of proteins in the oxidative phosphorylation pathway and the sirtuin signaling pathway. **C.** Disease and functional analyses for DEPs.

DEPs were further categorized using the IPA database to identify proteins with potential significant diagnostic values for GC. The pathway enrichment analysis indicated that DEPs were significantly enriched in oxidative phosphorylation, mitochondrial dysfunction, the sirtuin signaling pathway, and the tricarboxylic acid (TCA) cycle (Table S5). Of the 25 significant signaling pathways, 5 pathways (sirtuin signaling, interferon signaling, IL-8 signaling, neuroinflammation signaling, and inflammasome) with z-score > 0 were predicted to be activated in GC, whereas 20 pathways (z-score < 0) were predicted to be inhibited (Figure 4A). The most inhibited pathways were oxidative phosphorylation and the TCA cycle. Of particular interest, oxidative phosphorylation is potentially inhibited and the sirtuin signaling pathway activated in GC (Figure 4B). All proteins identified to be involved in oxidative phosphorylation were downregulated.

**Figure 5:**
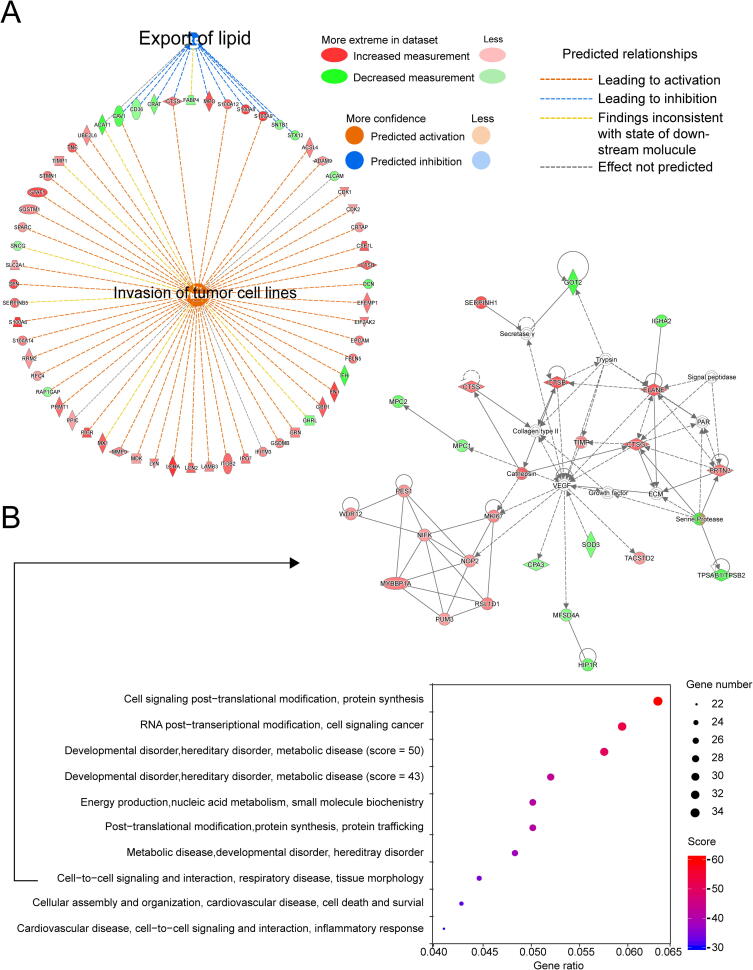
**Interaction network and biomarker analyses for DEPs** **A.** Interaction network analysis of tissue invasion by tumor cell lines and lipid export. **B.** Biomarker analysis for DEPs.

We performed disease and functional analyses for the abovementioned DEPs in IPA (Figure 4C), revealing that the most activated function was cell movement, including tissue invasion by various tumor cell lines. Tumor cell line proliferation, adhesion, and inflammatory responses were also found to be activated in tumors. The most inhibited function was lipid export. The network associated with regulation of tissue invasion by tumor cell lines and lipid export was connected by five proteins: ACAT1, CAV1, CTSS, S100A12, and S100A9 (Figure 5A).

Our biomarker analysis identified 72 potential biomarkers associated with cancer and gastrointestinal diseases, and the network analysis implicated 25 networks. Significant regulatory networks with scores > 30 were found associated with cell signaling, post-translational modification, protein synthesis, protein trafficking, energy production, nucleic acid metabolism, small molecule biochemistry, and cell-to-cell signaling and interaction (Figure 5B).

### Screening potential diagnostic markers in GC

**Figure 6:**
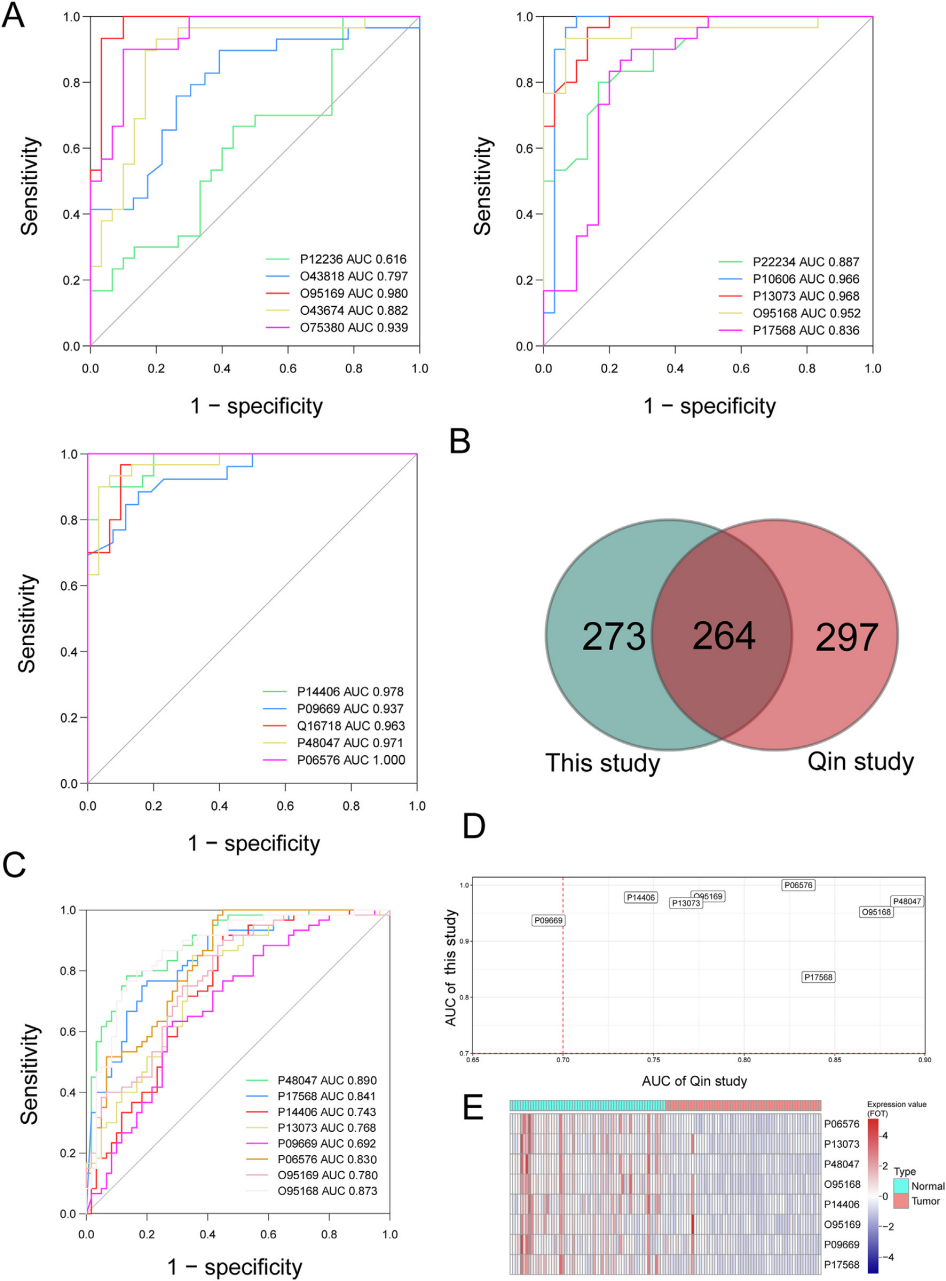
**Screening and validation of potential proteins as diagnostic markers for GC in the traning and testing sets** **A.** ROC curve for each hub protein in the training set. **B.** Venn diagram summarizing the number of DEPs between the traning (our study) and testing (Qin study [16]) sets. **C.** ROC curve for each shared hub protein in the testing set. **D.** Identification of potential and independent diagnostic proteins as biomarkers. **E.** Expression profile of significant shared hub proteins in the testing phase.

We next screened 15 hub proteins from the enriched canonical pathways, biomarker analyses, and the top ten protein–protein interaction networks (Table S6). The diagnostic performance of each protein was assessed using the ROC curve. Most proteins (13 out of 15) showed a high AUC value (> 0.800) between GC and healthy tissues, suggesting that these hub proteins might have discriminating potential as GC diagnostic markers (Figure 6A). Among these, NDUB8 (O95169) and CX7A2 (P14406) had AUC of 0.980 and 0.978, respectively. The 95% confidence interval (CI) of the AUC and *P* values compared with the reference line are shown in Table S7.

To test our newly identified proteins, we used an independently published cohort of 60 GC samples and matched healthy samples as the testing set [16]. A total of 561 DEPs including 345 upregulated and 216 downregulated proteins were identified in the testing set using the same filter criteria (Table S8). When compared with the results of our initial cohort (Figure 6B), 264 DEPs were shared between the two datasets, and 8 of the 15 hub proteins discussed above were differentially expressed in the testing set. As shown in Figure 6C, ATPO (P48047, AUC = 0.890) and NDUB4 (O95168, AUC = 0.873) were the two proteins with the highest predictive power. Proteins with AUC > 0.70 were considered as potential independent diagnostic biomarkers (Figure 6D). Only one protein (P09669) was excluded. Expression profiles of significant hub proteins found in the testing set are shown in Figure 6E.

### Establishment and validation of a four-protein signature

**Figure 7:**
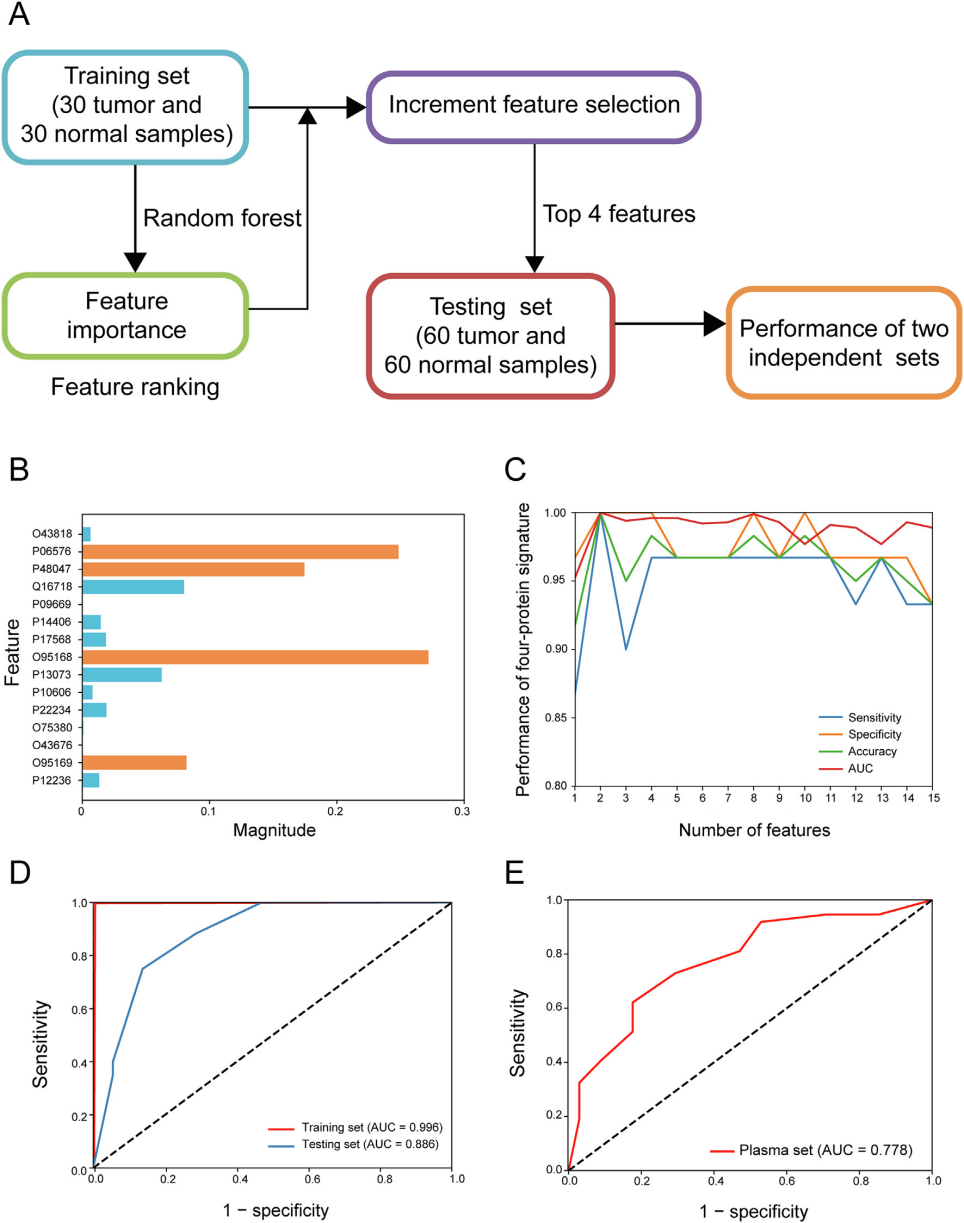
**Establishment and validation of a four-protein signature for the diagnosis ofGC** **A.** Design of the random forest model. **B.** Feature performance of each protein in the random forest model. **C.** Increment feature selection. AUC, sensitivity, specificity, and accuracy became constant and suitable when the four-protein signature was built. **D.** ROC curve for the four-protein diagnostic signature for the training set (red line) and testing set (blue line). **E.** ROC curve for the four-protein diagnostic signature for the plasma samples. AUC, area under curve.

Although the aforementioned results indicate that single proteins may hold significant discriminating potential, we investigated the possibility of building a multi-protein signature with increased diagnostic potential, sensitivity, and specificity. We used a random forest model (Figure 7A) including the performance of each protein. The best-performing proteins were found to be NDUB4 (O95168; encoded by *NDUFB4*), ATPB (P06576; encoded by *ATP5B*), ATPO (P48047; encoded by *ATP5O*), and NDUB8 (O95169; encoded by *NDUFB8*) (Figure 7B). After increment feature selection, AUC, sensitivity, specificity, and accuracy were found to be constant and suitable when the four-protein signature was built (Figure 7C). The AUC value of this protein signature in the training set was 0.996 (Figure 7D, red line) and the accuracy was 98.3%. Thus, we built a four-protein signature (*ATP5B*-*ATP5O*-*NDUFB4*-*NDUFB8*) with high diagnostic potential for GC.

To test the stability and diagnostic power of the four-protein signature, we analyzed the testing set consisting of 60 GC samples and matched healthy tissues [16] using LC-MS/MS. The AUC of the four-protein signature was 0.886 and its accuracy was 80%, suggesting that this protein signature had a high diagnostic value for GC (Figure 7D, blue line).

The aforementioned results were obtained from tumor and healthy tissues collected from patients and analyzed using LC-MS/MS. To further extend our study for clinical use and noninvasive detection, we used blood plasma samples from GC patients and healthy individuals to evaluate the potential diagnostic value of the protein signature for detecting GC using blood. We performed ELISA on plasma samples from 37 GC patients and 34 healthy controls for the validation phase. Our results showed that the protein signature had an AUC value of 0.778 and accuracy of 71.8% to distinguish GC tissues from healthy controls (Figure 7E).

**Figure 8:**
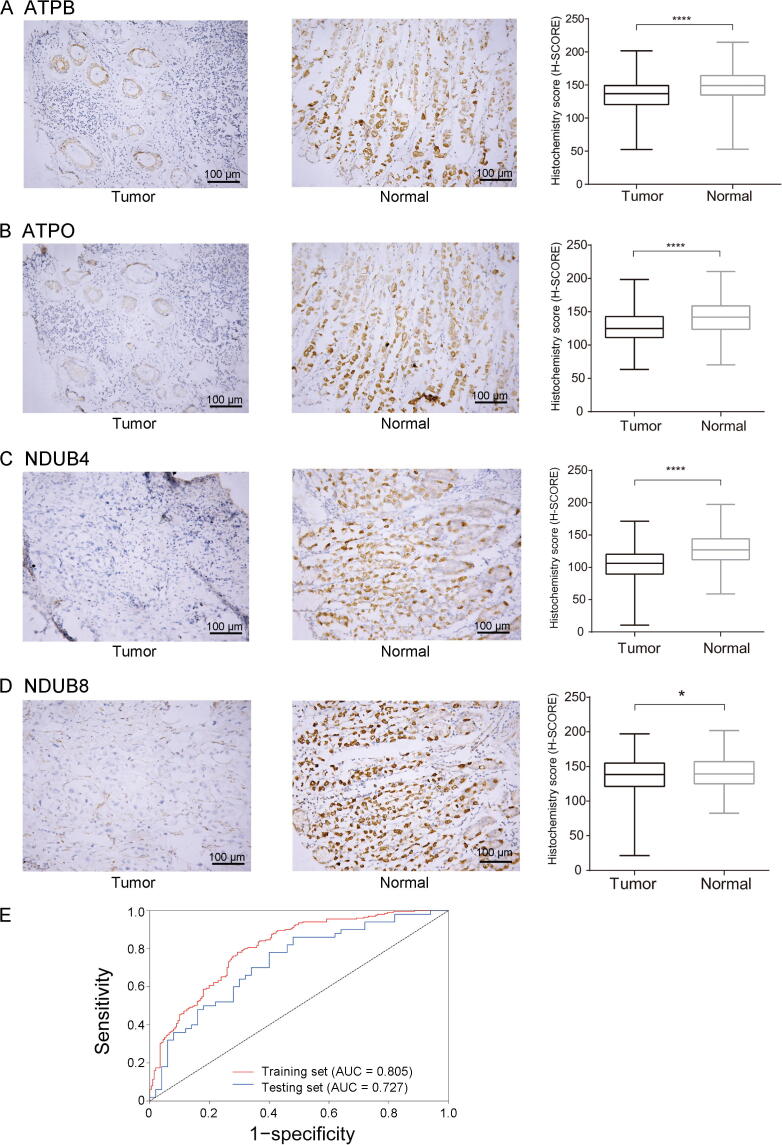
**Validation of a four-protein signature for the diagnosis ofGCin tissue microarray** **A.**–**D.** The immunohistochemical staining of ATPB (A), ATPO (B), NDUB4 (C), and NDUB8 (D) in tumor and normal GC tissues. Scale bars represent 100 μm. Each case was at ×200 magnification. *, *P* < 0.05; ****, *P* < 0.0001 (paired-samples *t*-test). **E.** ROC curve for the four-protein diagnostic signature for the tissue microarray.

Finally, we tested 251 pairs of GC tissues using tissue microarray. As shown in Figure 8A–D, we first determined differential expression patterns for the four proteins by immunohistochemical staining on tumor and normal tissues (*P* < 0.05). As this dataset included an adequate number of samples, we could divide the samples into internal training (80% samples) and testing (20% samples) sets. The AUC value for the internal training set was 0.805, with an AUC value of 0.727 for the internal testing set (Figure 8E). The *P* values compared with reference line for all datasets are shown in Table S9.

**Supplementary Figure S4:**
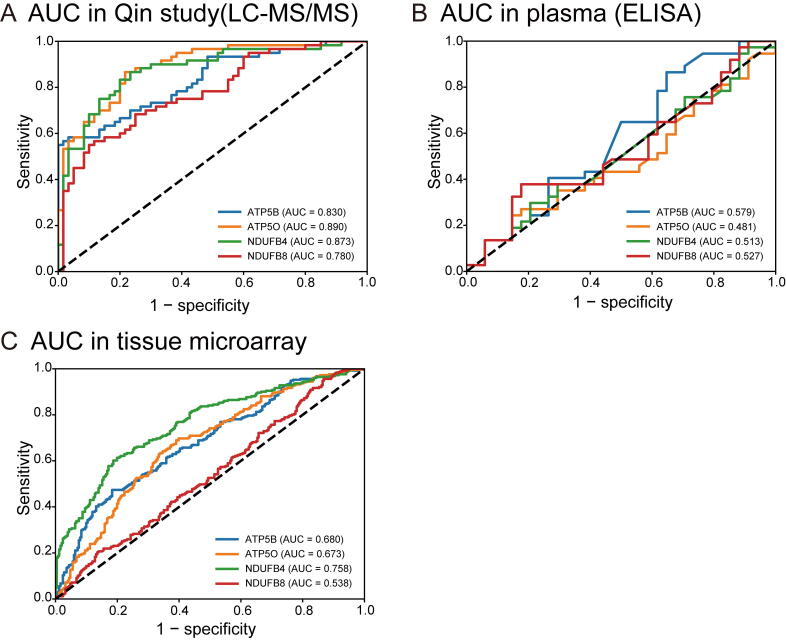
**The AUC values of each protein from the four-protein combination in these datasets**. **A**. The AUC of each protein in Qin’s study (LC-MS/MS). **B**. The AUC of each protein in plasma (ELISA). **C**. The AUC of each protein in tissue microarray. AUC, area under curve. ELISA, enzyme-linked immune sorbent assays. LC, liquid chromatography. MS, mass spectrometry.

At the same time, we tested the AUC values of each individual protein from the four-protein combination in aforementioned datasets. We found very small AUC values (Figure S4). In sum, our results suggest that this novel four-protein signature (*ATP5B*-*ATP5O*-*NDUFB4*-*NDUFB8*) has a high diagnostic power for GC.

## Discussion

In this study, we present a large-scale proteomic analysis of GC using label-free global proteome profiling based on 30 GC tissues paired with 30 healthy tissues. A four-protein (*ATP5B*-*ATP5O*-*NDUFB4*-*NDUFB8*) diagnostic signature was built using a random forest model. Our findings might help to understand the pathogenesis of GC and provide novel and specific therapeutic targets for this disease.

### Biomarkers and proteomics in cancer

Biomarkers are likely to exert an indispensable part in cancer diagnosis and treatment by enabling early detection and diagnosis [18]. Furthermore, the robust growth of quantitative proteomic methods has allowed researchers to analyze biomarkers for human tumors. For instance, proteomics has been used for biomarker discovery for colorectal cancer, including the role of protein phosphorylation and cancer stem cells [19]. Clinical proteomics has also been proven to be a promising tool for improving personalized medicine for colorectal cancer using blood, stool, and biopsy samples [20]. Moreover, MS-based proteomics has been used for drug discovery and development [21].

Other groups have also performed proteomic studies for GC. Huang et al*.* [22] performed a quantitative proteomic study using ten GC serum samples and healthy controls by tandem mass tags, and identified 594 serum DEPs with a cut-off value of 1.2 FC. The DEPs 1C12, PIGR, S10A8, AOC3, FHL1, GGCT, NCAM1, and SYNEM were also identified in our study. Liu et al*.* [23] performed label-free LC-MS/MS using GC tissues and healthy tissues from six patients and found 87 DEPs. Of these 87 proteins, ATPB, ATP4B, NDUB9, and NDUAD were also identified in our study. However, we used a more stringent screening criterion with a cut-off value of 2 FC and FDR < 0.05. The analysis of our testing set (60 pairs) revealed that 264 DEPs were shared between the two datasets. The DEPs identified by us are different from those of previous studies, likely due to the different platforms, quantitative methods, and screening criteria used. Therefore, more accurate analytical methods and a larger number of samples are necessary to confirm our findings.

Diagnostic test performance is often assessed by measuring ROC, AUC, sensitivity, and specificity. For an example, a label-free quantitative proteomic study was performed to diagnose periodontal diseases using saliva [24], and the authors found that 12 proteins presented the highest AUC (AUC 0.83–0.91) between healthy and diseased tissues. Yang et al. [25] used targeted proteomics coupled with immunoaffinity enrichment to investigate epithelial ovarian cancer samples and found that the combined AUC value for serum carbohydrate antigen 125 (CA125) and heat shock protein 27 was 0.88, which was significantly higher than that of CA125 alone. Jiang et al*.* [26] performed iTRAQ labeling and LC-ESI-MS/MS to evaluate a discovery group of four GC samples and four adjacent healthy tissues, and found an AUC value of 0.734 for GLS1 and GGCT co-expression, suggesting that the level of co-expression had a high clinical value as a diagnostic biomarker for early GC.

### IPA pathway analysis

Our results highlighted five activated pathways (sirtuin signaling, interferon signaling, inflammasome, IL-8 signaling, and neuroinflammation signaling) with z-score > 0 in GC. Among them, the sirtuin signaling pathway is the most active. Sirtuins are members of the class III histone deacetylase family [27], and mammalian sirtuins are classified into seven groups (SIRT1-7) [28]. Sirtuin signaling pathways can modulate stem cell functions which is of crucial vitality to normal embryonic development as well as adult tissue homeostasis [29]. In cancer, sirtuins are implicated in producing cancer cells capable of self-renewal and differentiation, resulting in tumor growth. Two previous studies also confirmed the role of sirtuins in stimulating epithelial-mesenchymal transition [30], [31]. The interferon signaling pathway and other innate immune signaling pathways in tumor cells were shown to be determinants of treatment response and resistance [32]. These pathways may serve as an alternative immunotherapeutic strategy in GC [33], [34], and our results suggest that these activated pathways exert an indispensable part in the pathogenesis of GC.

The most inhibited pathways in GC were oxidative phosphorylation and the TCA cycle. This is consistent with another report on GC [14]. A recent study has shown that cancer cells exhibit significant metabolic changes in mitochondrial dynamics and function [35]. Tumor growth is regulated by the TCA cycle and oxidative phosphorylation in mitochondria. According to the study of Warburg Effect [36], cancer cells are likely to increase energy metabolism via aerobic glycolysis instead of oxidative phosphorylation. Therefore, the regulation of energy metabolism via oxidative phosphorylation and the TCA cycle tends to be inhibited in GC, with aerobic glycolysis stimulated.

### The four proteins of the GC signature

Our study identifies a four-protein signature (*ATP5B*-*ATP5O*-*NDUFB4*-*NDUFB8*) with diagnostic value for GC. Of these, *ATP5B* has not been reported upon in previous studies on GC, however, it was found to be downregulated in clear cell renal cell carcinoma [37]. In glioblastoma, *ATP5B* mRNA levels were significantly higher in tumor cells than in healthy brain blood vessels, and microvascular proliferation was significantly higher [38]. *ATP5O* was reported as one member of eight mitochondrial genes including *NDUFS5*, *VDAC3*, *ATP5O*, *IMMT*, *MRPL28*, *COX5B*, *MRPL52*, and *PRKDC*, which generated a compact gastric mitochondrial gene signature for predicting tumor progression and overall survival of GC patients [39]. *ATP5O* gene expression was also downregulated in clear cell renal cell carcinoma [37]. *NDUFB8* was found to be hypermethylated in glioblastoma [40]. However, *NDUFB4* has been rarely reported upon in human cancers.

*ATP5B* and *ATP5O* encode two ATP synthases, and *NDUFB4* and *NDUFB8* encode two members of the NADH dehydrogenase (ubiquinone) 1 beta sub-complex. Mitochondrial dysfunction is common in cancer, and mitochondrial electron transport chains are often affected in carcinogenesis [41], [42]. Mitochondrial dysfunction is involved in cancer cell metabolism, apoptosis, and autophagy. Using antibiotics as anticancer drugs has been considered as potential anticancer therapy [43]. Therefore, targeting mitochondrial alterations might be a promising strategy for the development of tools for GC diagnosis, prognosis, and treatment.

### The performance of the four-protein signature in plasma

The performance of our four-protein signature was found to be better when measured in tissues (AUC = 0.996 and 0.886) than in plasma (AUC = 0.778). A major reason for this might be due to the stability of different experimental tests including LC/MS-MS and ELISA. A second reason might be attributed to the complexity of the multi-step process for analyzing blood plasma samples from tumor and healthy controls. However, the blood plasma test is still of value. Moreover, as described above, our protein signature is closely associated with energy metabolism. Therefore, this diagnostic protein signature may be applicable to other cancers in addition to GC.

### The differences between our and Qin’s studies

Recently, Qin’s group presented a dataset providing information on proteomics of gene products and mutations in cancer driver genes from 84 diffuse-type GC patients [16]. They divided the patients into three molecular subtypes that provided a wealth of information on diffuse GC signaling pathways and demonstrated the benefits of proteomic analysis in cancer molecular subtypes. In contrast to Qin’s study, our project focuses on MS-based proteomics and bioinformatic algorithms to screen biomarkers and models with diagnostic value in GC. In our study, we identified hub proteins with high diagnostic power in distinguishing tumors and normal controls. We further built a four-protein (*ATP5B*-*ATP5O*-*NDUFB4*-*NDUFB8*) diagnostic signature using a random forest model then verified it with GC tissues as well as with two independent datasets of plasma and tissue microarray. Our study identified potential biomarkers and may help increase our understanding of GC pathogenesis and provide novel and specific diagnostic targets for this cancer.

As shown in our multiple rounds of testing, our narrowed-down four-protein signature had high diagnostic power between tumor tissues and healthy controls, suggesting its potential use as a novel clinical biomarker for GC. However, further large-scale validation studies are necessary to confirm this finding.

## Conclusion

In summary, this study increased our understanding of GC pathogenesis and identified potential biomarkers to provide novel and specific therapeutic targets for this cancer.

## Materials and methods

### Selection of specimens and clinical information

Thirty tumor specimens and 30 matched healthy tissues were obtained from 30 GC patients following surgical resection at the First Hospital of China Medical University (Shenyang, China). The stain of tissue sections was made through hematoxylin and eosin to evaluate tumor purity by a certified pathologist in the hospital. Tumor samples consisting of at least 60% cancer cells were retained for further analysis (Figure S1A; Table S1). Each sample was collected within 30 min after surgical resection, cleaned, transferred to sterile freezing tubes, and cryopreserved in liquid nitrogen until further use. All samples were staged according to the seventh edition of The American Joint Committee on Cancer staging system.

In the testing phase, the diagnostic value of candidate proteins was estimated using the plasma of 71 individuals (37 GC patients and 34 healthy controls). In brief, 2 mL of the overall peripheral venous blood achieved the collection from every sample and then shifted onto a purple-top EDTA tube. The separation of plasma samples was made following a two-phase centrifugation protocol (3000 rpm for 5 min at 4 °C, and 12,000 rpm for 15 min at 4 °C) within 4 h after collection, and then the abovementioned samples were held in RNase/DNase-free tubes (Catalog No. MCT-150-C, Axygen, Union City, CA) and stored at −80 °C for further use.

### Protein extraction and tryptic digestion

GC tissues were ground into powder in liquid nitrogen and suspended in an ice-cold lysis buffer [8 M urea (Catalog No. V900119-500G, VETEC, Shanghai, China), 5 mM dithiothreitol (DTT; catalog No. D9163-5G, Sigma, Oakville, Canada), 1% (v/v) protease inhibitor cocktail (Catalog No. 539134, Merck, Darmstadt, Germany), 3 μM trichostatin A (TSA; catalog No. V900931-5MG, VETEC), 50 mM nicotinamide (NAM; catalog No. N0636-500G, Sigma, Oakville, Canada)] based on occasional sonication. The centrifugation of cell lysates was made to 12,000 *g* at 4 °C for the duration of 10 min; then came the collection of resulting supernatants. Total protein concentration was measured from lysates using the 2-D Quant kit (Catalog No. 80-6483-56, GE Healthcare, Pittsburgh, PA) abiding by manufacturer’s directions. Precipitation of proteins was then made with 15% trichloroacetic acid (TCA; catalog No. T4885-1KG, Sigma, Shanghai, China) for 4 h at 4 °C; the resulting precipitate got washed for three times with cold (−20 °C) acetone. The dried protein pellets were resuspended within 100 mM tetraethylammonium bromide (TEAB; catalog No. T7408-500ML, Sigma, Buchs, Switzerland) and then digested with trypsin (Catalog No. V5111, Promega, Madison, WI) on the condition of an enzyme-to-substrate rate of 1:50 for 12 h at 37 °C. Reduction of peptides with DTT and alkylation with iodoacetamide (Catalog No. V900335-5G, VETEC) were performed in the dark. Complete digestion was ensured by performing a second digestion with trypsin at the enzyme-to-substrate rate of 1:100 for 4 h at 37 °C.

### Peptide fractionation using HPLC

The sample fractionation was performed by high pH reverse-phase HPLC based on an Agilent 300Extend C18 column (particle size, 5 μm; ID, 4.6 mm; length, 250 mm. Catalog No. Agilent 1260 Infinity, Agilent Technologies, Palo Alto, CA). In short, peptides were first divided into 80 fractions using a gradient of 2%–60% acetonitrile (ACN) in 10 mM ammonium bicarbonate (Catalog No. V900254-500G, VETEC) pH 10 for 80 min, and then they were combined into multiple fractions (10 for label-free proteome) and dried using vacuum centrifugation (Eppendorf, Hamburg, Germany).

### LC-MS/MS analysis

For label-free proteomic analysis, peptides were dissolved in 0.1% formic acid (FA; catalog No. 94318-50ML-F, Fluka, St. Louis, MO), loaded onto a reversed-phase pre-column (Catalog No. Acclaim PepMap 100, Thermo-Fisher Scientific, Waltham, MA), and separated using a reversed-phase analytical column (Catalog No. Acclaim PepMap RSLC, Thermo Fisher Scientific). The elution gradient used was 5%–10% of solvent B (0.1% FA in 98% ACN) for 4 min, 10%–23% of solvent B for 36 min, 23%–35% of solvent B for 12 min, 35%–80% of solvent B for 4 min, and 80% of solvent B for 4 min. Elution was performed at a constant flow rate of 300 nl/min using an EASY-nLC 1000 ultra-performance liquid chromatography (UPLC) system. Peptides subjected to post-translational modifications were separated in a similar way and analyzed using a Q Exactive TM Plus hybrid quadrupole-Orbitrap mass spectrometer (ThermoFisher Scientific).

Peptides got in the subordination of a nanoelectrospray ionization source followed by MS/MS using a Q Exactive TM Plus (ThermoFisher Scientific) mass spectrometer coupled online to a UPLC system. Detection of intact peptides was performed by using Orbitrap at a resolution of 70,000. Selected peptides for MS/MS were fragmented using a normalized collision energy of 30 for MS/MS; ion fragments were detected using Orbitrap at a resolution of 17,500. Using a data-dependent program that alternates between one MS scan and 20 MS/MS scans, the top 20 precursor ions above the threshold ions in the MS survey scan are subjected to different dynamic exclusion. For proteome detection, the ion count threshold and dynamic exclusion used were 1E4 and 30.0 s, respectively.

### Analysis of global proteomics data

The resulting MS/MS data were analyzed by the MaxQuant with an integrated Andromeda search engine (version 1.4.1.2). The search for tandem mass spectra was implemented to a SwissProt human database (downloaded on August 2015) concatenated with a reverse decoy database. Trypsin/P was defined as the cleavage enzyme allowing up to two missing cleavages. For proteomic analysis, the first search range was set to 5 ppm for precursor ions, and the main search range was set to 5 ppm and 0.02 Da for fragment ions. The carbamidomethylation of cysteines was defined as the fixed modification, and the oxidation on methionine was defined as the variable modification. The quantification method used was LFQ, the FDR was adjusted to < 1%, and the minimum score for modified peptides was > 40.

### Identification of DEPs

To identify the proteins differentially expressed between tumor and healthy tissues, DEPs were defined as meeting the following criterion: |Log_2_ FC| > 1 and FDR < 0.05. The heatmap of expression profiles was drawn using the *pheatmap* package in R language. GO enrichment analysis was performed to assess the functional biological role of DEPs. For GO term enrichment, including MF, biological process, and cellular component, a two-tailed Fisher’s exact test was used to determine the enrichment of DEPs against all proteins identified in-house using a Perl script. Terms with an FDR < 0.05 were considered significant. The graphical summary of GO results was drawn using the *ggalluvial* R package.

### IPA

The molecular and biological functions of DEPs were analyzed using IPA (Qiagen, https://www.qiagenbioinformatics.com/products/ingenuity-pathway-analysis/) [44]. This analytical tool includes canonical pathway analysis, interaction network analysis, disease and functional analysis, and a biomarker filter. The two statistical indicators of IPA used were the *P* value and z-score. *P* < 0.05 was considered statistically significant, and the z-score was calculated using an internal algorithm and IPA standard. The molecular interaction was activated when the z-score was > 0 and inhibited when the z-score was < 0.

### Screening and validation of diagnostic markers using ROC analysis

The ROC curve was used to further select potential biomarkers with diagnostic power by determining the specificity and sensitivity of each protein, and the AUC was used to estimate the diagnostic value. The ROC curve was drawn using the *pROC* R package.

For the testing cohort, a proteomic dataset from 60 GC patients obtained using label-free analysis was used [16]. Raw data of all samples were downloaded, and the same search engine MaxQuant as well as the same filter criterion were used to identify DEPs. Based on these results, ROC analysis was conducted to test the diagnostic power of potential hub proteins in the testing cohort.

### Analysis using a random forest model

To further build a multi-protein signature with diagnostic power, the significant hub DEPs were used as attributes, and an analysis using a random forest model was performed in-house using a python script. In brief, this method was used together with 10-fold cross-validation for the training set to build a four-protein signature. Moreover, the diagnostic value of this model was verified using ROC analysis. Sensitivity, specificity, accuracy, and AUC were used to determine predictive values.

In the testing phase, based on the expression of these four proteins, an analysis using a random forest model was performed to prove the accuracy of the diagnostic value of the four-protein signature.

### Validation of the four-protein signature using ELISA

The diagnostic power of the four-protein signature was validated using 71 plasma samples (37 from GC patients and 34 from healthy controls). In brief, human plasma ATPB, ATPO , NDUB4, and NDUB8 levels were measured using commercial ELISA kits (Catalog No. JL46945 for ATPO, catalog No. JL46944 for ATPB, catalog No. JL47136 for NDUB4, and catalog No. JL47139 for NDUB8, Shanghai Jianglai Biotech, Shanghai, China) based on the manufacturer’s instructions.

### Validation of the four-protein signature using tissue microarray

Immunohistochemistry was performed using 5 µm thick tissue microarray sections as previously described [45]. After dewaxing in xylene and rehydration through graded alcohol, sections were placed in 3% hydrogen peroxide to block endogenous peroxidase. For antigen retrieval, sections were boiled in citrate/EDTA buffer (citrate buffer for ATPO, ATPB, and NDUB4, catalog No. ZLI-9065, ZSGB-BIO, Beijing, China; EDTA buffer for NDUB8, catalog No. R20904, Shanghai Yuanye Bio-Technology, Shanghai, China). After blocking with goat serum (Catalog No. SP-9001 for ATPB and NDUB8, ZSGB-BIO; catalog No. SP-9002 for ATPO and NDUB4, ZSGB-BIO), sections were incubated overnight with anti-ATPO/anti-ATPB/anti-NDUB4/anti-NDUB8 antibodies (anti-ATPO: 1:5000, catalog No. ab110276, Abcam, Cambridge, UK; anti-ATPB: 1:5000, catalog No.33031, SAB, College Park, MA; anti-NDUB4: 1:1000, catalog No. ab110243, Abcam; anti-NDUB8: 1:8000, catalog No. ab192878, Abcam) at 4 °C. Sections were then further incubated with reagents from the SP IHC Kit (Catalog No. SP-9001 for ATPB and NDUB8, ZSGB-BIO; catalog No. SP-9002 for ATPO and NDUB4, ZSGB-BIO) with 15 min for each reagent. Sections were visualized using DAB (Catalog No. ZLI-9010, ZSGB-BIO) and Hematoxylin.

Slides were analyzed with Panoramic MIDI digital scanner (Catalog No. 3DHISTECH, Budapest, Hungary). Quantitative image analysis was performed by the QuantCenter software using histochemistry score (H-SCORE).

### Statistical analysis

The SPSS software version 19.0 (SPSS, Inc., Chicago, IL) was used to perform statistical analyses. The differences in DEP expression between GC tissues and matched healthy tissues were determined based on the Student’s *t*-test. ROC and AUC were used to assess the diagnostic value of each candidate protein and the four-protein signature. Two-tailed *P* values < 0.05 were considered statistically significant.

## Ethical statement

The research was carried out abiding by the principles established by the Declaration of Helsinki and was approved by the Human Research Ethics Committee of China Medical University (Shenyang, China). A written acquainted consent was obtained from all patients.

## Data availability

The MS proteomics data were deposited in the iProX database (iProX: IPX0001590000), and are publicly accessible at at https://www.iprox.org/.

## CRediT author statement

**Yongxi Song:** Conceptualization, Methodology, Writing - original draft. **Jun Wang:** Conceptualization, Methodology, Formal analysis, Writing - original draft. **Jingxu Sun:** Resources, Writing - review & editing, Visualization. **Xiaowan Chen:** Methodology, Formal analysis, Resources. **Jinxin Shi:** Writing - review & editing. **Zhonghua Wu:** Resources. **Dehao Yu:** Resources. **Fei Zhang:** Resources, Writing - review & editing. **Zhenning Wang:** Writing - review & editing, Project administration. All authors read and approved the final manuscript.

## Competing interests

The authors declare no conflicts of interest.
